# Similar polyethylene wear between cemented and cementless Oxford medial UKA: a 5-year follow-up randomized controlled trial on 79 patients using radiostereometry

**DOI:** 10.1080/17453674.2018.1543757

**Published:** 2018-12-10

**Authors:** Kristian Horsager, Frank Madsen, Anders Odgaard, Claus Fink Jepsen, Lone Rømer, Per Wagner Kristensen, Bart L Kaptein, Kjeld Søballe, Maiken Stilling

**Affiliations:** a Department of Clinical Institute, Aarhus University, Aarhus, Denmark;; b Department of Orthopedics, Aarhus University Hospital, Aarhus, Denmark;; c Department of Orthopaedics, Herlev-Gentofte Hospital, Copenhagen University Hospital, Hellerup, Denmark;; d Department of Radiology, Aarhus University Hospital, Aarhus, Denmark;; e Department of Orthopedics, Vejle Hospital, Vejle, Denmark;; f Department of Orthopedic Surgery, Biomechanics and Imaging Group, Leiden University Medical Center, Leiden, Netherlands

## Abstract

Background and purpose — Hydroxyapatite (HA)-coated implants have been associated with high polyethylene wear in hip arthroplasties. HA coating as a promoter of wear in knee arthroplasties has not been investigated. We compared the wear-rate of the polyethylene bearing for cemented and cementless HA-coated Oxford medial unicondylar knee arthroplasties (UKA). Secondarily, we investigated whether wear-rates were influenced by overhang or impingement of the bearing.

Patients and methods — 80 patients (mean age 64 years), treatment-blinded, were randomized to 1 of 3 Oxford medial UKA versions: cemented with double-pegged or single-pegged femoral component or cementless HA-coated with double-pegged femoral component (ratios 1:1:1). We compared wear between the cemented (n = 55) and cementless group (n = 25) (ratio 2:1). Wear, impingement, and overhang were quantified between surgery and 5-year follow-up using radiostereometry. Clinical outcome was evaluated with the Oxford Knee Score.

Results — The mean wear-rate for patients without bearing overhang was 0.04 mm/year (95% CI 0.02–0.07) for the cemented group and 0.05 mm/year (CI 0.02–0.08) for the cementless group. The mean difference in wear was 0.008 mm/year (CI –0.04 to 0.03). No impingement was identified. Half of the patients had medial bearing overhang, mean 2.5 mm (1–5). Wear increased by 0.014 mm/year for each mm increment in overhang. The mean Oxford Knee Score was 39 for the cementless group and 38 for the cemented group at the 5-year follow-up.

Interpretation — The wear-rates were similar for the 2 fixation methods, which supports further use of the cementless Oxford medial UKA. However, a caveat is a relatively large 95% CI of the mean difference in wear-rate. Component size and position is important as half of the patients presented with an additional increase in wear-rate due to medial bearing overhang.

Polyethylene (PE) wear is an important factor for failure of knee arthroplasties (Lonner et al. 1999, Røkkum et al. 1999, Sharkey et al. 2002). The failure mechanism is particle-induced osteolysis and subsequent late aseptic loosening or accelerated catastrophic wear (Kadoya et al. 1998, Harris 2001, Naudie et al. 2007). The Swedish Knee Arthroplasty Register from 2016 reported that 11% of all unicondylar knee arthroplasty (UKA) revisions were performed due to PE wear (Sundberg et al. 2016). The Oxford medial UKA is designed to reduce PE wear with a fully congruent mobile bearing dynamically linked between a spherical femoral component and a flat tibial surface (O’Connor and Goodfellow 1996). The PE bearing has previously been found with very low wear-rates of less than 0.03 mm/year, if the bearing moves freely with no impingement against surrounding structures (Psychoyios et al. 1998, Kendrick et al. 2010a, 2010b).

Traditionally, the Oxford medial UKA has been inserted by use of bone cement. In 2004, a cementless hydroxyapatite (HA)-coated design was introduced to improve fixation properties, eliminate cementing errors, and reduce duration of surgery (Tai and Cross 2006, Pandit et al. 2009, Liddle et al. 2013, Kendrick et al. 2015). However, HA coating has been associated with high wear-rates and unacceptable revision rates in several studies of total hip arthroplasties (THA) (Hallan et al. 2006, Kim et al. 2006, Gottliebsen et al. 2012). Revision studies have found HA particles embedded in the articulating surface of the PE liner and argue that HA is the most probable cause of third-body wear (Bloebaum et al. 1994, 1997, Morscher et al. 1998, Røkkum and Reigstad 1998). To our knowledge, HA coating as a promoter of PE wear in knee arthroplasties has not been investigated.

Radiostereometry (RSA) and recent developments of advanced model-based software allow for the measurement of in-vivo PE wear in the Oxford medial UKA with high accuracy (Van IJsseldijk et al. 2011, 2014).

The primary endpoint of this study was a comparison of PE wear between cemented and cementless Oxford medial UKA, which is a secondary objective of the randomized controlled trial (RCT). We hypothesized that there would be no difference in the wear-rate. Secondarily, we investigated whether wear-rates were influenced by bearing overhang or impingement of the bearing against the vertical wall.

## Patients and methods

### Participants

**Figure 1.**
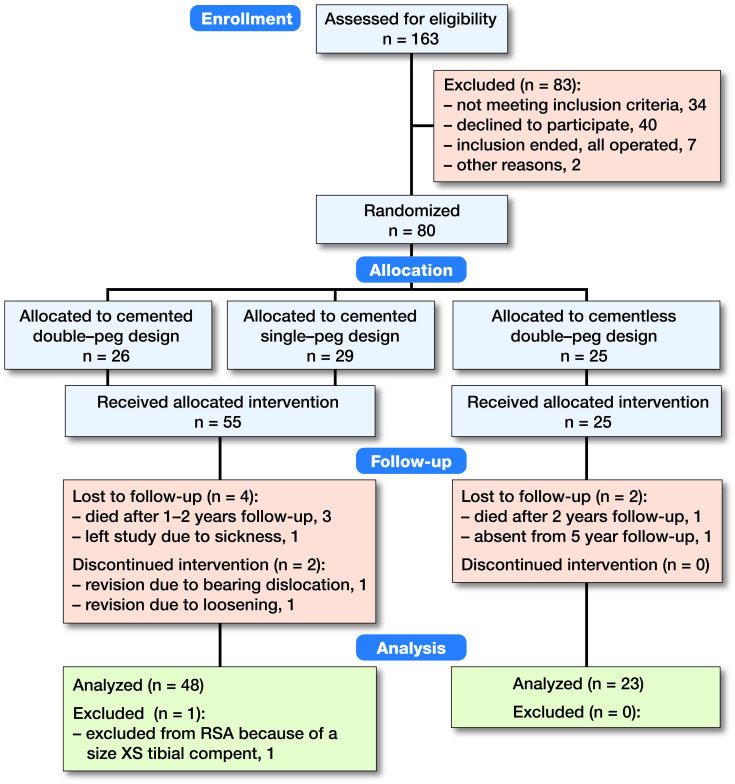
Consort flow chart. All available data were used in the statistical analysis. A complete dataset was collected for 48 patients in the cemented group and for 23 patients in the cementless group. Demographic data at baseline were comparable between the 2 groups (Table 2). All patients were followed with RSA, conventional radiographs, and Oxford Knee Score over the 5-year follow-up period.

This patient- and observer-blinded multicenter RCT was carried out at 2 hospitals in Denmark (Aarhus University Hospital and Vejle Hospital). Patients with knee pain and isolated medial compartment osteoarthritis in telos-stress radiographs and otherwise suitable for treatment with Oxford medial UKA were assessed for eligibility to participate. The surgeons enrolled all patients. Inclusion criteria were medial compartment osteoarthritis, patients >18 years old, and informed consent. Exclusion criteria are outlined in Table 1 (see Supplementary data). 163 consecutive patients were evaluated for eligibility and 83 were excluded (Figure 1). All patients received a phase 3-alpha Oxford medial UKA with ArCom ultra-high molecular weight polyethylene-bearing (ZimmerBiomet, Warsaw, IN, USA). The implant was inserted with bone cement (Refobacin Bone Cement R, ZimmerBiomet, Warsaw, IN, USA) or press-fit cementless HA-coated fixation during 2009–2011. The cementless Oxford UKA used in this study (2nd generation) had 0.75 mm titanium plasma spray and was coated with 55 μm HA.

**Table 1.** Exclusion criteria

1	Neuro- or vascular disease in the affected leg
2	Extension deficit >10°
3	Preoperative maximal flexion <100°
4	Symptomatic patellar OA**^a^**
5	Insufficient anterior cruciate ligament (ACL)
6	Lateral compartment OA
7	Preoperatively templated for a size XS or XL femoral component**^b^**
8	Osteoporosis
9	Continuous vitamin K antagonist treatment
10	Fracture sequelae in the knee
11	Previous extensive surgery
12	Metabolic bone disease
13	Rheumatoid arthritis
14	Hormonal substitution for postmenopausal symptoms
15	Steroid treatment
16	Non-Danish citizens
17	Insufficient command of the Danish language
18	Dementia
19	Misuse of drugs or alcohol
20	Serious psychiatric disease
21	Disseminated malignant disease
22	Systemic hip or back condition
23	Poor dental status
24	Participation in another study

**^a^**All patients were screened for patellar OA with patellar radiographs

**^b^**XS or XL femoral computer aided design (CAD) models were not available for analysis.

Exclusion criteria are given from Clinicaltrials.gov, NCT00679120 (Stilling and Søballe).

**Table 2.** Patient demographics at baseline

	Cemented	Cementless
	(n = 55)	(n = 25)
Men: women, n	30: 25	18: 7
Right: left, n	32: 23	11: 14
Age, years ^a^	63 (9) [47–81]	65 (10) [38–81]
Weight, kg ^a^	87 (13) [67–121]	88 (14) [61–110]
Surgeons involved, n	7	5

**^a^**mean (SD) [range]

Patients were randomized in blocks of 12 with sequentially numbered envelopes that were opened during surgery. Randomization was performed 3-armed with equal distribution, as the cemented group could receive a double-pegged or single-pegged femoral component. The cementless femoral component was only double-pegged. This allowed a 3-way comparison of the femoral-component migration, which is a primary endpoint of the RCT (unpublished) in which the present study is nested. This is not of interest with respect to PE wear and therefore we considered the randomization 2-armed with a 2:1 ratio for the cemented (n = 55) and cementless group (n = 25).

### Sample size

Sample size and power were not calculated for PE wear. The sample size was based on the primary migration outcome (unpublished data). A detailed description of all sub-study purposes is given at ClinicalTrials.gov (NCT00679120).

### RSA examinations

RSA examinations were taken immediately postoperatively, and at 6, 12, 24, and 60 months of follow-up. Double examinations for assessment of precision were conducted on all patients at the 6 months follow-up in accordance with the ISO 2013 RSA standards (Table 3, see Supplementary data).

**Table 3.** Precision of minimal joint space width (mJSW) measurements and the femorotibial contact-point location

Item	Mean	1.96 x SD
mJSW measurements, mm	0.01	0.12
Femorotibial contact point, mm		
medio-lateral	0.05	1.16
anterior-posterior	0.21	2.51

The table presents the mean and 1.96 x SD of the difference between double exposures.

**Figure 2.**
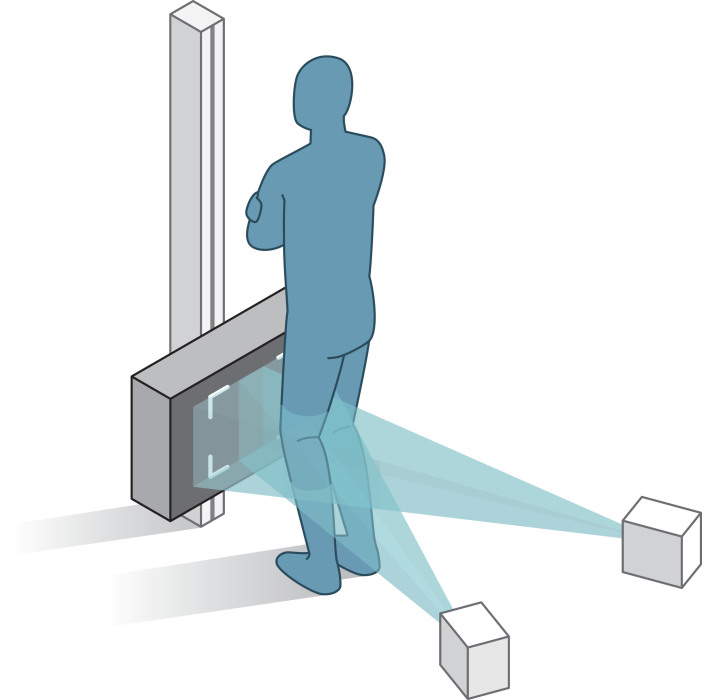
This figure outlines the set-up of the RSA examinations. To standardize the set-up, a rectangular foam support was applied between the leg and calibration-box. Patients were also asked to move their center of gravity over the operated leg to make sure that the prosthetic knee joint was loaded.

During the initial study period (2009–2014), examinations were performed with the Arcoma system (Arco Ceil model 0070-S, Växjö, Sweden). After January 2014, a direct digital dedicated stereo X-ray system was used (AdoraRSA-suite, NRT, Aarhus, Denmark). For both RSA systems, the X-ray tubes were positioned horizontally with an angle of 40° between tubes, and a source-to-image-detector distance of 160 cm. The uniplanar calibration-box (CarbonBox 14, Medis Medical Imaging Systems, Leiden, Netherlands) was equipped with 2 digital image detectors: the Adora system used wireless CXDI-70C detectors (Canon, Tokyo, Japan) and the Arcoma system used FCR Profect CS detectors (Fujifilm, Vedbaek, Denmark). During the examinations, patients were standing and facing the calibration box, with the knee loaded and slightly flexed (10–20°) (Figure 2). The resolution of the RSA images was 203 pixels per inch.

### Model-based RSA analysis

**Figure 3.**
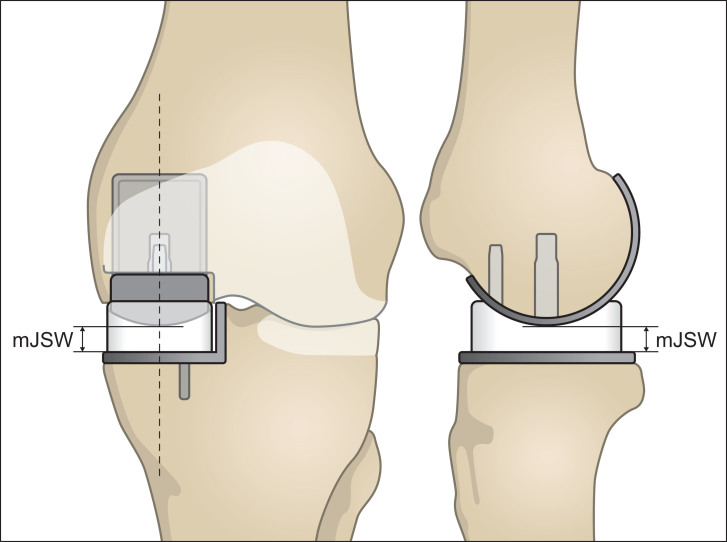
The frontal view to the left outlines the measurement of the minimal joint space width (mJSW). The dotted line represents the sagittal cross-sectional view presented to the right. The mJSW reflects the bearing thickness and the projected dotted line on the tibial component represents the femorotibial contact point. This allows the estimation of PE wear and the position of the bearing. Overhang is seen when the bearing exceeds the outline of the tibial plateau. Impingement can be identified if the bearing slides against the vertical wall (see frontal view).

The stereoradiographs were analyzed with Model-based RSA (Version 4.01, RSAcore, Leiden, Netherlands) (Kaptein et al. 2003). Wear was evaluated from the minimal joint space width (mJSW). The mJSW is an indirect measure of the bearing thickness, as it defines the shortest distance between the spherical femoral component perpendicular to the flat tibial component (Figure 3) (Van IJsseldijk et al. 2011, Horsager et al. 2018). The relative difference in the measured mJSW over the 5-year follow-up represents the combined upper- and lower-surface PE wear.

Bearing overhang and impingement against the vertical wall were approximated using the femorotibial contact point, as the bearing is not visible on the stereoradiographs. The femorotibial contact point was defined from the projected mJSW line and reflects the center position of the bearing (Figure 3). This is allowed because of the spherical design of the femoral component and the fully congruent bearing. Overhang was noted when the distance between the femorotibial contact point and the most medial, anterior, or posterior edge of the tibial component was less than the corresponding dimensions of the bearing. Impingement against the vertical wall was noted when the mediolateral distance between the femorotibial contact point and the vertical wall was less than half the width of the bearing (Horsager et al. 2018).

The measurements assume that the bearing is kept parallel with the vertical wall, and that no rotation occurs. Bearing overhang and impingement were only used for further analysis if this exceeded the precision for the corresponding contact-point location (Table 3, see Supplementary data). For each patient, the measured maximal bearing overhang at all follow-up times was used in the statistical analysis. If impingement was measured, it would indicate an error-full mJSW, as it would imply lift-off. Any mJSW measurements with impingement would be excluded in the statistical analysis.

### Clinical outcome

Clinical outcome was evaluated using the Oxford Knee Score (OKS). OKS scores for baseline, 5-year follow-up, and the difference between baseline and 5-year follow-up (D OKS) are presented (Table 4, see Supplementary data).

**Table 4.** Oxford Knee score. Values are mean (95% CI)

Type	Baseline OKS	5-year OKS	D OKS ^a^
Cemented	26 (24–27)	38 (36–40)	13 (10–15)
Cementless	23 (21–26)	39 (37–42)	16 (13–19)

**^a^**D OKS describes the change in clinical outcome from baseline to 5-year follow-up. There was no significant difference in D OKS between the cemented and cementless group (p = 0.1). The sample decreased from n = 54 to 46 for the cemented group and n = 25 to 24 for the cementless group for 5-year OKS and D OKS.

### Statistics

A linear mixed model for repeated measurements was used to evaluate the wear-rate based on the mJSW measurements from baseline to 5-year follow-up using a linear relationship. 2 models were computed: (1) a crude model including the fixed-effects of fixation type (cemented vs. cementless), bearing overhang and their appropriate interaction with time, and (2) an “adjusting” model adding the fixed effects of sex, baseline weight, and D OKS.

Included interaction terms with time were justified by visual inspection of the plotted model residuals against the interaction variable. Interaction terms for “fixation type and time” and “medial bearing overhang and time” were included. Patient ID was included as a random factor and the development over time was identified by the random slope. Model diagnostics were validated by visual inspection of residuals and fitted values. Gaussian distributions were checked using QQ plots. P-values were derived from the model output, which allowed a test of the main null hypothesis and sub-hypothesis. Sex, weight, and clinical outcome were not tested for significance. The effect of the adjusting variables was evaluated from the change in wear-rate between the crude model and adjusting model. A 2-sample Satterthwaite t-test was used to compare the D OKS. The 95% confidence interval (CI) was calculated for all wear-rate measurements and all OKS scores. The statistics were performed in collaboration with the Biostatistical Advisory Service at Aarhus University, Denmark. P-values <0.05 were regarded as statistically significant. Graphs and analysis were generated using Stata 14.0 (StataCorp LP, College Station, TX, USA).

### Ethics, registration, funding, and potential conflict of interest

Approvals were obtained from the local ethics committee (M-20070258; d. 15/01/2008) and Data Protection Agency (2008-41-2104; d. 28/03/2008) and the study was conducted in agreement with the Helsinki II declaration. The study was registered at ClinicalTrials.gov (NCT00679120). The study was financially supported by Biomet Inc. The authors have no conflict of interest to declare.

## Results

**Figure 4.**
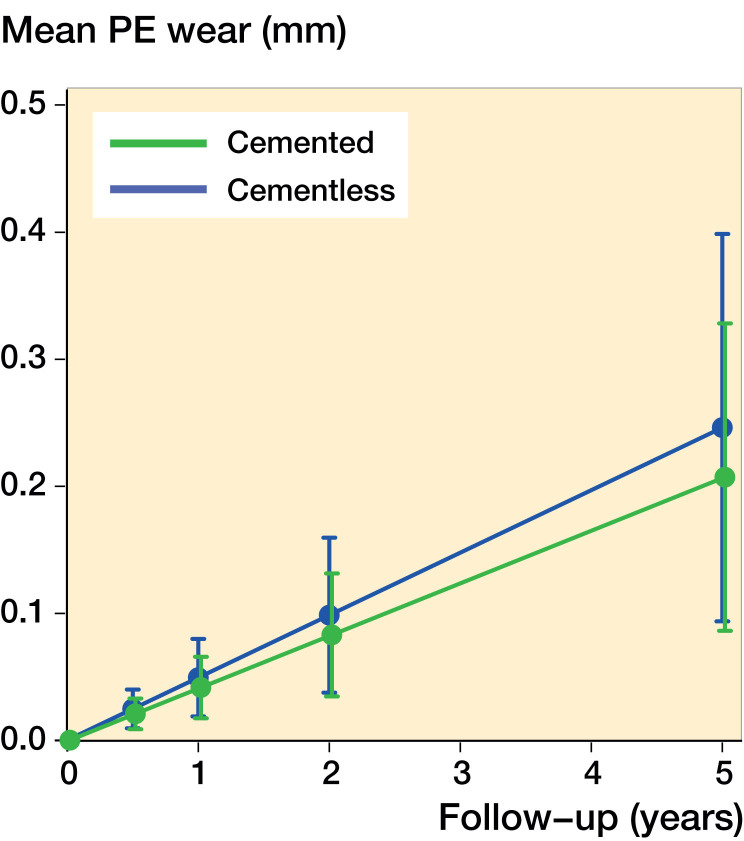
The linear wear-rate of the cemented and cementless Oxford UKA with 95% CI from the linear mixed model.

The mean wear-rate was 0.04 mm/year (CI 0.02–0.07) for the cemented design and 0.05 mm/year (CI 0.02–0.08) for the cementless design, if no bearing overhang or impingement was measured (crude model). The mean difference in wear-rate was –0.008 mm/year (CI –0.04 to 0.03). Sex, weight, and clinical outcome (adjusting variables) had minimal effect on the wear-rate, as the adjusting model produced equivalent results: no change was seen for the cementless design and the wear-rate of the cemented design increased to 0.05 mm/year (CI 0.02–0.07). There was no statistically significant difference between the cemented and cementless fixation method (p = 0.6) (Figure 4). An extreme patient outlier was identified from the residuals, and a sensitivity analysis excluding the outlier from the model generated the same results. Also, a sub-analysis showed equal wear-rates for the single- and double-pegged design for the cemented Oxford medial UKA.

**Figure 5.**
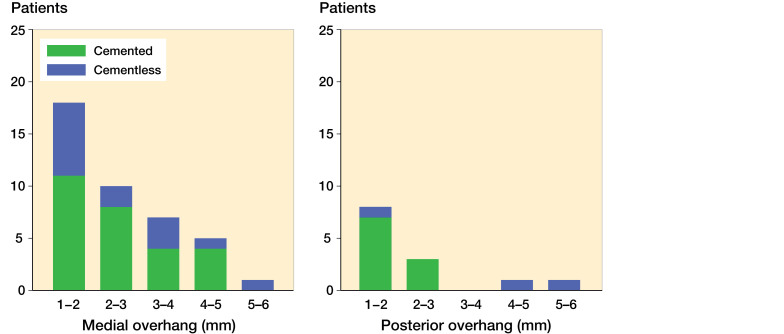
Distribution plots for the extent of bearing overhang. Medial overhang is shown to the left and posterior overhang is shown to the right.

**Figure 6.**
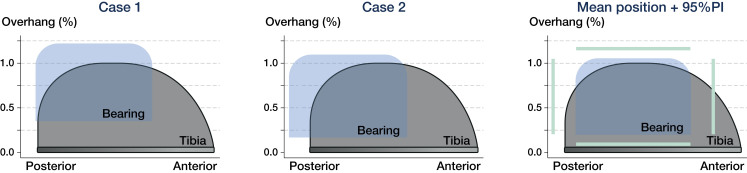
This figure visualizes a “top view” of the PE bearing and tibial component of the Oxford Medial UKA. The figures are based on the assumption that the bearing is kept parallel with respect to the vertical wall. Extreme cases of bearing overhang are visualized in Case 1 and 2. The mean bearing position for all patients with 95% prediction interval is visualized in the right panel. Case 1 represents the patient with 5 mm medial overhang and case 2 represents the patient with 5 mm posterior bearing overhang. The graphs are computed using the femorotibial contact point and the exact ratio between the size of the bearing and tibial component.

Bearing overhang ranging from 1 to 5 mm was seen in 41 patients on the medial side, mean 2.5 mm (1–5 mm) and in 14 patients for the posterior edge of the tibial component, mean 2 mm (1–5 mm) (Figures 5 and 6). None of the patients had anterior bearing overhang or impinged the bearing against the vertical wall.

The effect of medial bearing overhang was statistically significant, and the wear-rate increased by 0.014 mm/year (CI 0.004–0.025) for each mm increment of overhang (p = 0.01), resulting in wear-rates ranging from 0.064 to 0.12 mm/year. This did not change after adjusting for weight, sex, and clinical outcome. The effect of posterior overhang on wear-rate was not included in the model, as there was no sign of interaction with time in the plotted residuals. The D OKS scores were similar between the cemented and cementless group (Table 4, see Supplementary data).

## Discussion

To our knowledge, this is the first study to compare PE wear for cemented and HA-coated cementless knee arthroplasties. The concern of HA coating as a promoter of PE wear is mostly derived from studies of THAs (Bloebaum et al. 1994, 1997, Morscher et al. 1998, Røkkum and Reigstad 1998, Stilling et al. 2009, Gottliebsen et al. 2012). Our results contradict this expectation, as the cemented and cementless Oxford medial UKA presented similar wear-rates of 0.04–0.05 mm/year.

Another finding was the increase in wear-rate ranging from 0.064 to 0.12 mm/year for half of the patients due to medial bearing overhang.

HA may only cause third-body wear if it enters the joint. This is hypothesized to happen during insertion, from chemical dissolution and from mechanical abrasion of the coating due to loosening of the implant and lack of initial stability (Morscher et al. 1998, Røkkum and Reigstad 1998, Røkkum et al. 1999, Duffy et al. 2004). Full osseointegration is believed to limit the dissociation of HA-particles to the joint and protect the fixation-interface from wear debris—a phenomenon called “Sealing-effect” (Kadoya et al. 1998, Morscher et al. 1998, Rahbek et al. 2005). The HA-coated parts of the Phase 3 Oxford medial UKA were completely covered by bone and the fixation interface stabilizes within the first 6 months (Kendrick et al. 2015). These are optimal conditions for a successful sealing effect.

It may be speculated that only little force is applied to the HA coating during implantation of an Oxford UKA. This should limit the risk of initial delamination of the HA coating compared with the forces generated for the insertion of a press-fit cup. Moreover, the fully congruent mobile bearing causes compressive loads and limits shear forces, which may help reduce debonding of the HA coating before full osseointegration (Müller and Patsalis 1997, Horsager et al. 2017, 2018). Overall, the Oxford medial UKA exhibit several features that in theory reduce the dissociation of HA particles to the joint, which thus limits the potential of third-body wear. This could explain similar wear-rates for both fixation methods. However, the 95% CI of the mean difference in wear-rate was relatively large and cannot exclude all clinically relevant differences in wear-rates, as no threshold value for wear has been established.

The mean wear-rate for the cemented Oxford UKA (with phase 1 and 2 bearings) is well documented and ranges from 0.026 mm/year to 0.07 mm/year (Argenson and O’Connor 1992, Psychoyios et al. 1998, Price et al. 2005, Kendrick et al. 2010a, 2010b). This is comparable to our results (phase 3 bearings). However, Kendrick et al. (2010b) and Psychoyios et al. (1998) found that retrieved bearings with no sign of abnormal macroscopic wear or impingement had very low wear-rates of 0.01 mm/year. It was concluded that well-functioning bearings, which move freely with no impingement against surrounding structures, such as osteophytes or the implant itself, should achieve wear-rates <0.03 mm/year (Kendrick et al. 2010b). To account for this, we approximated whether the bearings were “well-functioning,” by measuring if the bearings impinged against the vertical wall or exceeded the outline of the tibial plateau (bearing overhang). Bearing overhang does not necessarily reflect a poorly functioning bearing. Yet it does lower the surface contact area and increase the risk of edge-loading and impingement (Figure 6). The measured wear-rate of 0.05 mm/year for the cementless design and 0.04 mm/year for the cemented design (no overhang or impingement) represents a “well-functioning” bearing. This is somewhat higher than the expected wear-rate of <0.03 mm/year. However, it does not statistically violate the statement given the 95% confidence interval.

None of the implants showed impingement. This could be due to the standing RSA examinations, as impingement could be induced by knee motion (Horsager et al. 2018).

Medial bearing overhang ranging from 1 to 5 mm was measured in half of the patients. We found an additional increase in wear-rate of 0.014 mm/year for each mm increment in overhang, which leads to a relatively high wear-rate ranging from 0.064 to 0.12 mm/year (Figure 5). This is in line with Kendrick et al. (2010b) and Psychoyios et al. (1998), who found wear-rates >0.1 mm/year, if the retrieved bearings had signs of impingement. The measured posterior bearing overhang could be expected to have the same effect, although this was not evident. To our knowledge, this is the first study to report the presence, extent, and effect of bearing overhang. The finding outlines the importance of component alignment and careful surgical technique.

The use of the femorotibial contact point as an indirect measure of impingement and bearing overhang raises some limitations. The method assumes that the bearing is kept in the center of the femorotibial contact point. Rotation of the bearing and impingement from osteophytes or soft tissue is undetectable. Still, this is probably the best we can achieve as the bearing is not visible on the stereoradiographs. The measurements were further limited from the standing RSA examinations, as the mobile bearing moves throughout motion (Horsager et al. 2018).

Some concern has been addressed with the risk of overestimation of wear with shorter follow-up periods, as the PE bearing is stated to creep in the first 6 months (Glyn-Jones et al. 2008, Kendrick et al. 2010b). We did not identify initial creep of the bearing, as the residuals for the wear measurements did not conflict with the linear model. This does not imply that creep does not occur—only that it is probably insignificant.

The proportion of loss to follow-up was equal for the cemented and cementless group and below the critical limit of 20%, which minimizes the risk of bias (Dettori 2011). The bias should be further minimized from the linear mixed model, as it handles missing data effectively and allowed the use of all available data (Krueger and Tian 2004).

Sample size and power were not calculated, since wear of the PE bearing was a secondary effect parameter and therefore the results can be a type II error. Our study is also susceptible to multiplicity issues, as several effect parameters have been studied in the RCT. However, this is the largest PE wear study performed on the Oxford medial UKA (Argenson and O’Connor 1992, Psychoyios et al. 1998; Price et al. 2005, Kendrick et al. 2010a, 2010b). If a difference exists in PE wear between well-functioning cemented and cementless Oxford medial UKAs, it would be small and most likely clinically insignificant.

The patient’s weight, sex, and clinical outcome did not seem to influence the wear-rate. This may indicate that obese patients or a poor clinical outcome are not associated with high wear-rates in the UKA.

We believe our study provides solid data for the wear-rate of clinically successful Oxford medial UKAs, due to the precise loaded state-of-the-art in-vivo RSA measurements and sufficient follow-up time of 5 years. The results may support the continued use of the cementless HA-coated Oxford medial UKA and indicate that the most significant contributing factor for PE wear is based on the kinematics and position of the PE bearing. In addition, although the Oxford medial UKA design is ingenious, it is technical demanding and may require more strict alignment for optimal conditions.

Furthermore, our results indicate that the wear-rate of the Oxford medial UKA is generally higher than expected and may approach linear wear-rates measured for non-congruous fixed-bearing UKAs, which have been reported as 0.15 mm/year for the St George Sled UKA (Ashraf et al. 2004). Volumetric wear could be comparable due to the fully congruent design of the Oxford medial UKA (Burton et al. 2012).

In summary, we found similar wear-rates for the cemented and HA-coated cementless Oxford medial UKA in a randomized study design, which supports continued use of the cementless Oxford medial UKA. However, a caveat is a relatively large 95% CI of the mean difference in wear-rate. Half of the patients presented with an additional increase in wear-rate due to medial bearing overhang, which emphasizes the importance of careful component positioning. Overall, the measured wear-rate was low, but slightly higher than expected for clinically successful Oxford medial UKA—especially if medial bearing overhang was present.

### Supplementary data

Tables 1, 3, and 4 are available as supplementary data in the online version of this article, http://dx.doi.org/10.1080/17453674.2018.1543757

## Supplementary Material

Supplemental Material

## Notes

KS, MS, AO, FM, and PWK designed the study. FM, AO, CFJ, PWK, LR, and MS collected the data. KH, MS, and BLK analyzed the data and performed the statistics. KH, BLK, MS, FK, PWK, and AO helped with data interpretation. KH wrote the initial manuscript draft. All authors revised and approved the final manuscript.

The authors thank orthopedic surgeons Dan Blohm, Bjørn Gottlieb, and Anders Lamberg for surgeries on some of the patients. They thank Rikke Mørup, Lone Løvgren Andersen, and Henriette Appel for their valued assistance in patient management and data collection. ZimmerBiomet, the Danish Rheumatoid Association, and the A.P. Møller Foundation kindly provided financial support for data analyses.

*Acta* thanks Stephan Maximilian Röhrl and Leif Ryd for help with peer review of this study.
