# Supplementary material for: Similar polyethylene wear between cemented and cementless Oxford medial UKA: a 5-year follow-up randomized controlled trial on 79 patients using radiostereometry
Source: Acta Orthop. 2018 Dec 10;90(1):67–73. doi: 10.1080/17453674.2018.1543757 (PMC6366473; doi:10.1080/17453674.2018.1543757)
Supplement: Supplemental Material [file IORT_A_1543757_SM9560.pdf]

Supplementary data

Table 1. Exclusion criteria

1	Neuro- or vascular disease in the affected leg
2	Extension deficit > 10°
3	Preoperative maximal flexion < 100°
4	Symptomatic patellar OA [a]
5	Insufficient anterior cruciate ligament (ACL)
6	Lateral compartment OA
7	Preoperatively templated for a size XS or XL femoral component [b]
8	Osteoporosis
9	Continuous vitamin K antagonist treatment
10	Fracture sequelae in the knee
11	Previous extensive surgery
12	Metabolic bone disease
13	Rheumatoid arthritis
14	Hormonal substitution for postmenopausal symptoms
15	Steroid treatment
16	Non-Danish citizens
17	Insufficient command of the Danish language
18	Dementia
19	Misuse of drugs or alcohol
20	Serious psychiatric disease
21	Disseminated malignant disease
22	Systemic hip or back condition
23	Poor dental status
24	Participation in another study

[a] All patients were screened for patellar OA with patellar radiographs
[b] XS or XL femoral computer aided design (CAD) models were not available for analysis.
Exclusion criteria are given from Clinicaltrials.gov, NCT00679120 (Stilling and Søballe).

Table 3. Precision of minimal joint space width (mJSW) measurements and the femorotibial contact-point location

Item	Mean	1.96 x SD
mJSW measurements, mm	0.01	0.12
Femorotibial contact point, mm		
medio-lateral	0.05	1.16
anterior-posterior	0.21	2.51

The table presents the mean and 1.96 x SD of the difference between double exposures.

Table 4. Oxford Knee score. Values are mean (95% CI)

Type	Baseline OKS	5-year OKS	Δ OKS [a]
Cemented	26 (24–27)	38 (36–40)	13 (10–15)
Cementless	23 (21–26)	39 (37–42)	16 (13–19)

[a] Δ OKS describes the change in clinical outcome from baseline to 5-year follow-up. There was no significant difference in Δ OKS between the cemented and cementless group (p = 0.1). The sample decreased from n = 54 to 46 for the cemented group and n = 25 to 24 for the cementless group for 5-year OKS and Δ OKS.
